# C-Terminal Amination of a Cationic Anti-Inflammatory Peptide Improves Bioavailability and Inhibitory Activity Against LPS-Induced Inflammation

**DOI:** 10.3389/fimmu.2020.618312

**Published:** 2021-02-05

**Authors:** Lulu Zhang, Xubiao Wei, Rijun Zhang, Matthew Koci, Dayong Si, Baseer Ahmad, Henan Guo, Yanfei Hou

**Affiliations:** ^1^ State Key Laboratory of Animal Nutrition, College of Animal Science and Technology, China Agricultural University, Beijing, China; ^2^ Prestage Department of Poultry Science, College of Agriculture and Life Sciences, North Carolina State University, Raleigh, NC, United States; ^3^ School of Pharmaceutical Sciences, Tsinghua University, Beijing, China

**Keywords:** C-terminal amination, cellular uptake, bioavailability, Toll-like receptor, lipopolysaccharide neutralization, NF-кB signaling

## Abstract

Lipopolysaccharide (LPS) has been implicated as a major cause of inflammation and an uncontrolled LPS response increases the risk of localized inflammation and sepsis. While some native peptides are helpful in the treatment of LPS-induced inflammation, the use of these peptides is limited due to their potential cytotoxicity and poor anti-inflammatory activity. Hybridization is an effective approach for overcoming this problem. In this study, a novel hybrid anti-inflammatory peptide that combines the active center of Cathelicidin 2 (CATH2) with thymopentin (TP5) was designed [CTP, CATH2 (1–13)-TP5]. CTP was found to have higher anti-inflammatory effects than its parental peptides through directly LPS neutralization. However, CTP scarcely inhibited the attachment of LPS to cell membranes or suppressed an established LPS-induced inflammation due to poor cellular uptake. The C-terminal amine modification of CTP (CTP-NH_2_) was then designed based on the hypothesis that C-terminal amidation can enhance the cell uptake by increasing the hydrophobicity of the peptide. Compared with CTP, CTP-NH_2_ showed enhanced anti-inflammatory activity and lower cytotoxicity. CTP-NH_2_ not only has strong LPS neutralizing activity, but also can significantly inhibit the LPS attachment and the intracellular inflammatory response. The intracellular anti-inflammatory effect of CTP-NH_2_ was associated with blocking of LPS binding to the Toll-like receptor 4-myeloid differentiation factor 2 complex and inhibiting the nuclear factor-kappa B pathway. In addition, the anti-inflammatory effect of CTP-NH_2_ was confirmed using a murine LPS-induced sepsis model. Collectively, these findings suggest that CTP-NH_2_ could be developed into a novel anti-inflammatory drug. This successful modification provides a design strategy to improve the cellular uptake and anti-inflammatory activity of peptide agents.

## Introduction

Lipopolysaccharide (LPS), a major component of the cell wall of gram-negative bacteria, has been implicated as a major cause of inflammation (1, 2). An uncontrolled LPS response gives rise to excessive localized inflammation, such as liver inflammation (3), and severe systemic responses to infection, such as sepsis (4). Hence, LPS control and clearance is critical for avoiding excessive inflammation and organ damage. Traditionally, antibiotics, such as polymyxin B, are therapeutically important in the treatment of LPS-induced inflammation (5). Regrettably, the development of polymyxin B as an anti-inflammatory drug has faced several obstacles, primarily attributed to undesirable side effects, such as neuro- and nephrotoxicity, hampering their clinical development (5). Therefore, there is an urgent need to identify and develop new drugs that possess improved pharmaceutical profiles and reduced adverse effects.

In recent years, some native bioactive peptides have been suggested as a promising strategy to develop new anti-inflammatory agents (6–8). A wide variety of organisms, such as mammals, insects, fish, amphibians and plants, secrete peptides as important immunomodulators (9). Many native peptides have been reported to have certain inhibitory activities against LPS-induced inflammation, such as LL-37 (10–13), Cathelicidin 2 (CATH2) (14), and Thymopentin (TP5) (15, 16). Among them, CATH2 and TP5 have displayed enormous potential in the treatment of LPS-induced inflammation (14–16).

CATH2 is a highly cationic (11^+^) chicken heterophil-derived peptide. It has been reported to have strong anti-inflammatory effects through LPS neutralization (14) and regulating the mRNA expression of proinflammatory cytokines, including IL-1β, IL-6, and TNF-α (14). Therefore, DEFB126 can prevent or attenuate LPS-induced inflammation.

TP5, the Arg32–Tyr36 fragment derived from thymopoietin, was found to exert its anti-inflammatory effect by inhibiting the transcription factor NF-κB and p38 signaling cascades (15–19). Besides, TP5 plays an important role in T-lymphocyte maturation and differentiation, thereby regulating immunity and the inflammatory response (20, 21). Overall, TP5 is used in the treatment of inflammatory diseases, such as infectious diseases, due to its anti-inflammatory activities and low cytotoxicity.

However, the development of CATH2 has been weakened by its potential cytotoxicity (22). TP5 has minimal cytotoxicity, but its development has been weakened by its short half-life, which decreases its efficacy and bioavailability (23, 24). As a simple and effective strategy that can combine the advantages of different native peptides (25, 26), hybridization has been put forward to improve the anti-inflammatory activity and physiological stability and reduce the undesirable cytotoxic effects of these native peptides (25). As previously reported, CATH2 (1–13) (14) exhibits robust anti-inflammatory activities. Therefore, to obtain a novel peptide with increased anti-inflammatory activity but minimal cytotoxicity, we designed a hybrid peptide (CATH2-TP5, CTP) by combing the active center of CATH2 [CATH2 (1–13)] with TP5. The new designed peptide, CTP, efficiently inhibited LPS-induced inflammation. However, CTP only suppress the LPS-induced inflammatory response when it interacted with LPS but did hardly attenuate an established LPS-stimulated inflammation, which was speculated to be a result of poor cellular uptake.

To overcome the difficulty of peptide access and entry into the cell, various methods have been employed. For instance, introduction of histidine residues (27, 28) or addition of D-amino acids (29, 30) may enhance peptide transmembrane delivery. Furthermore, peptide hydrophobicity is required for enhanced cellular uptake (31, 32) and C-terminal amidation has been reported to enhance the hydrophobicity of peptides (33). Therefore, we attempted to modify CTP to obtain a C-terminal amidated derivative peptide (CTP-NH_2_) to improve cellular uptake, intracellular distribution and consequently anti-inflammatory activity. The hydrophobicity of the peptide was characterized by retention time. *In vitro* experiments were performed to evaluate the cytotoxicity, anti-inflammatory effect, and anti-inflammatory mechanism of the derivative peptide. Furthermore, its anti-inflammatory effects were assessed through an LPS-induced murine model of sepsis.

## Materials and Methods

### Hybrid Peptide Design

The hybrid peptide CATH2-TP5 (CTP, RWGRFLRKIRRFRRKDVY) was constructed by combining the active center of CATH2 (RWGRFLRKIRRFRPKVTITIQGSARF) with TP5 (RKDVY). Primary sequence analysis of all the peptides was performed using ExPASy Proteomics Server: http://www.expasy.org/tools/protparam.html.

### Peptides Synthesis

The peptides CATH2, TP5, and CTP were synthesized in free C-terminal acid form, and CTP-NH_2_ was synthesized in amidated C-terminal acid form. The peptides were synthesized and purified by KangLong Biochemistry (Jiangsu, China). The purity of the peptides was determined by HPLC and mass spectrometry (MS). All the peptides had purities of 95% or greater. The peptides were dissolved in endotoxin-free water and stored at -80°C.

### The Retention Time of Peptides

The retention time of peptides on a reverse-phase matrix has been reported to be related to peptide hydrophobicity (34). Thus, the relative hydrophobicity of hybrid peptides in aqueous solution indicated that differences in hydrophobicity were reliably reflected by different HPLC retention times.

### Cell Culture

Mouse macrophage (RAW264.7) cells were cultured in Dulbecco’s modified Eagle’s medium (DMEM) (HyClone, Logan, UT, USA) containing 10% (v/v) fetal bovine serum (FBS) (Gibco, Foster, CA, USA) and 1% (v/v) penicillin/streptomycin (HyClone), at 37°C in a moist atmosphere (5% CO_2_, 95% air).

### Cell Viability Assay

The viability of peptide-treated RAW264.7 cells was determined using a Cell Counting Kit-8 (CCK-8) Assay Kit (Dojindo) (35). RAW264.7 cells were plated in 96-well plates at a density of 3×10^4^ cells/ml in 100 μl DMEM overnight. The cell culture medium was then supplemented with fresh medium containing candidate peptides in a series of concentrations, and the plates were incubated for another 24 h or 72 h. Each well was incubated with 10 μl CCK-8 solution for 4 h in the dark. Afterwards, the absorbance at 450 nm was measured using a microplate reader. Cell viability was determined by:

$$Cell\;viability\;\left(\%\right)=\left(\text{OD}450\frac{\text{sample}}{\text{OD}450\left(\text{control}\right)}\right)\times 100\%$$

### Anti-Inflammatory Assay in the RAW264.7 Cell Line

RAW264.7 cells were treated with or without 10 μg/ml peptides for 30 min before the addition of 100 ng/ml LPS (*E. coli*, O55:B5, Sigma-Aldrich, Germany) and further incubation for 12 h at 37°C After treatment, the concentrations of TNF-α, IL-6, and IL-1β in the cell supernatants were assessed.

To identify the mechanisms underlying the anti-inflammatory effects, three different types of treatments were performed as follow.

#### Peptide Pretreatment

Briefly, 10 μg/ml peptide was added to the cell culture medium and incubated with cells for 1 h at 37°C Afterwards, the RAW 264.7 cells were washed with PBS and cultured with fresh medium containing 100 ng/ml LPS for 12 h at 37°C. After treatment, TNF-α, IL-6, and IL-1β secretion in the cell supernatants was assessed.

#### Peptide Neutralized LPS

The peptides (10 μg/ml) were incubated with 100 ng/ml LPS directly for 1 h at 37°C. RAW 264.7 cells were stimulated with these mixtures for 12 h at 37°C. Afterwards, the TNF-α, IL-6 and IL-1β levels in the cell supernatants were assessed.

#### Peptide Post-Treatment

Briefly, 100 ng/ml LPS was added to the cell culture medium and incubated with cells for 1 h at 37°C. Afterwards, the RAW 264.7 cells were washed with PBS and cultured with fresh medium containing 10 μg/ml peptides for 12 h at 37°C. Afterwards, the TNF-α, IL-6, and IL-1β levels in the cell supernatants were assessed.

### Confocal Laser-Scanning Microscopy

RAW264.7 cells were treated with N-terminus FITC-labeled peptides at 10 μg/ml for 24 h at 37°C; in the dark. Then, the RAW264.7 cells were rinsed with PBS three times, fixed with paraformaldehyde and washed with PBS. The cell nuclei were stained with DAPI (diluted 1:500 in PBS) (Sigma, USA) for 5 min, and the cells were washed with PBS. The above cells were spread on a glass slide, fixed and observed with a Leica TCA sp5 confocal microscope (Germany).

### Flow Cytometry

RAW264.7 cells were stained with 10 μg/ml N-terminus FITC-labeled peptides at 37°C in the dark for 24 h. Afterwards, the RAW264.7 cells were harvested and rinsed with PBS five times. The average FITC intensity in the cells was measured *via* flow cytometry.

### Neutralization of LPS

The neutralization of LPS by the peptides was assessed through a quantitative Chromogenic End-point Tachypleus Amebocyte Lysate (CE TAL) assay using a QCL-1000 kit (XIAMEN BIOENDO TECHNOLOGY CO., China). LPS (*E. coli*, O111:B4, Sigma-Aldrich, USA) at a final concentration of 1.0 U/ml was incubated with various concentrations of the peptides (0 to 64 μg/ml final concentration) at 37°C for 15 min. Afterwards, the mixtures were incubated with TAL assay reagent at 37°C; for 6 min, and the absorbance was measured at 540 nm.

### Western Blotting

RAW264.7 cells plated at a density 1.8 × 10^6^ cells/ml were incubated with LPS (100 ng/ml) at 37°C for 1 h. After that, the cells were washed extensively with PBS before being treated with CTP-NH_2_ for 3 h at 37°C, followed by lysis of the cells. Cytoplasmic and nuclear protein fractions were obtained using NR-PER Nuclear and Cytoplasmic Extraction Reagents (Thermo Fisher Scientific Inc., New Zwaland). The protein concentrations were assessed with a CA kit (KeyGEN Biotech. Nanjing, China) according to the manufacturer’s instructions. Afterwards, total protein (40 μg protein/lane) was separated on 10% SDS-PAGE gels and then transferred to PVDF membranes (Bio-Rad). Next, the membranes were blocked with 5% non-fat dried-milk containing 0.05% TBST and then immunoblotted with specific primary antibodies against IKK-β, p-IKK-β, IкB-α, p-IкB-α, NF-кB (p65), p-NF-кB (p-p65), and β-actin (Santa Cruz, CA, USA). After being washed with TBST, the membranes were incubated with HRP-conjugated secondary antibodies (HuaAn, Hangzhou, China). A ChemiDoc MP Imaging System (Bio-Rad, Hercules, CA, USA) was used to quantify the density of the specific proteins.

### Molecular Dynamics Simulation

The initial 3D structure of the peptide was generated through Chimera software. To assess the binding affinity of with the TLR4/MD-2 complex, the relevant crystallographic structure of the Toll-like receptor 4/myeloid differentiation factor 2 (TLR4/MD-2) complex was retrieved from PDB (PDB code: 2Z64). The missing hydrogen atoms were added under pH 7.0 conditions by Maestro (36). The protein-protein docking server RosettaDock (version 3.5) was used to predict and assess interactions between the peptides and the binding target TLR4/MD-2 complex. To filter the best docking conformers, we selected conformations with the lowest binding energy and a greater number of hydrogen bonds.

The best binding poses of the peptide with TLR4/MD-2 was subjected to Molecular Dynamics (MD) simulation under AMBER14 (37, 38). The protein systems were treated with GAFF and FF14SB and were solvated under the periodic boundary conditions in a cubic box with the TIP3P water model (39). Na^+^ and Cl^–^ atoms were added to mimic physiological conditions and neutralize each system before the production. The system was first minimized with 5,000 steps by the conjugate gradient algorithm, followed by heating gradually in 100 ps. Subsequently, the volume of the system was adjusted at constant pressure (NPT: the number of particles, pressure of the system and temperature of the system remained constant) (40). After that, the equilibrated structures were simulated under a constant number, volume, and temperature (NVT) for 60 ns.

Based on the 300 snapshots extracted from the last 40 ns of the equilibrated MD simulation, the binding energy was calculated based on the molecular mechanics Poisson-Boltzmann accessible surface area (MM-PBSA) method (41). The Particle-mesh Ewald (PME) method was used to calculated the long-range electrostatic interactions of the system (42).

### Surface Plasmon Resonance (SPR)

SPR assays were performed using a Biacore X100 instrument (GE Healthcare, Pittsburgh, PA, USA). PBS containing 0.05% Tween 20 was used as the running buffer. The running buffer was continuously passed into the reaction chamber at 30 μl/min. Immobilization of CTP-NH_2_ on the chip surface was performed according to an amino coupling protocol. To obtain the sensorgrams of the interactions between the peptides and TLR4/MD-2 complex, a range of peptide concentrations (0, 1.25, 2.5, 5, and 10 mM) were analyzed. Running buffer was injected into the empty channel as a reference. To regenerate the chip surface at the end of each experiment, 10 mM Gly-HCl buffer (pH 2.5) was injected. ProteOn manager software (version 2.0) was used to analyze the experimental data. The binding curves were processed for the start injection alignment and baseline. A reference-subtracted sensorgram was then fitted to the curves describing a homogeneous 1:1 model. Data from the protein surfaces were grouped together to fit the association kinetic rate constant (*K*a) and the dissociation rate constant (*K*d). The equilibrium dissociation constant (*K*D) for the peptide-TLR4/MD-2 interaction was calculated as follows:

KD=Kd/Ka

### Animal Model

Male C57/BL6 mice (6–8 weeks of age) were purchased from Charles River (Beijing, China). The mice were maintained in a specific-pathogen-free (SPF) environment at 22 ± 1°C with relative 55 ± 10% humidity during the experiments. The assays were performed in conformity with the laws and regulations for live animal treatments at China Agricultural University.

The mice were randomly distributed into three groups (n = 12 each): control, LPS (*E. coli*, O111:B4, Sigma-Aldrich, USA) treatment, and CTP-NH_2_ pretreatment followed by LPS treatment (CTP-NH_2_ + LPS). For the first 7 days, CTP-NH_2_ (10 mg/kg mouse weight) was injected intraperitoneally once daily. Meanwhile, an equal volume of sterile saline was injected into mice in the control and LPS-treated groups. On day 7, LPS (10 mg/kg mouse weight) was injected intraperitoneally into mice in the LPS and CTP-NH_2_ + LPS groups to establish the sepsis animal model. The control group was intraperitoneally injected with an equal volume of saline. Sixteen hours after the LPS injection, the mice were euthanized by cervical dislocation, and samples of the intestine were collected for analysis.

### Histopathology and Immunohistochemistry

The mouse liver tissues were fixed in 4% paraformaldehyde, embedded in paraffin and cut into 5-μm-thick sections using an RM2235 microtome (Leica, Germany). The sections were stained with hematoxylin-eosin (H&E), and a DM3000 microscope was used to acquire images. LPS-induced liver injury was evaluated according to the following four categories: alveolar congestion, hemorrhage, neutrophil infiltration into the airspace or vessel wall, and thickness of alveolar wall/hyaline membrane formation. The liver injury score was graded on a 0- to 4-point scale: 0, no injury; 1, up to 25% injury in the field; 2, up to 50% injury in the field; 3, up to 75% injury in the field; 4, diffuse injury (43).

For immunohistochemical analysis, the sections were blocked with PBS containing 1% w/v BSA for 1 h at room temperature. Afterwards, the sections were incubated with anti-CD177^+^ antibody (1:100; Santa, USA). Samples were washed with PBS followed by incubation with horse-radish peroxidase (HRP)-conjugated rabbit anti-goat IgG (1:100; JIR, USA) at 4°C for 1 h. Subsequently, slides were stained with 3,3’-diaminobenzidine (DAB; DAKO, USA) and then counterstained with Harris hematoxylin. Finally, the samples were dehydrated in an alcohol gradient (70-100%) and cleared in xylene. All slides were mounted in neutral balsam.

### ELISA

The levels of tumor necrosis factor (TNF)-α, interleukin (IL)-6, and IL-1β in cell culture supernatants and the levels of TNF-α, IL-6, and IL-1β in the serum of mice were detected using commercial ELISA kits (eBioscience, San Diego, USA) according to the manufacturer’s instructions. The levels of serum alanine amino transferase (ALT) and aspartate amino transferase (AST) were detected using commercial reagent kits (Nanjing Jiancheng Bioengineering Institute, Nanjing, China). The activity of myeloperoxidase (MPO) in the liver of mice was detected using an ELISA kit (Boster, Wuhan, China) according to the manufacturer’s instructions.

### Statistics

All the data are expressed as the mean values ± standard deviation of at least three independent experiments. Statistical comparisons were carried out with *Student’s* t test using GraphPad Prism v6 software (La Jolla, California). Significance was claimed at p values ≤ 0.05; NS: p > 0.05, *: p ≤ 0.05, **: p ≤ 0.01, ***: p ≤ 0.001, and ****: p ≤ 0.0001.

## Results

### Peptide Design and Characterization

As shown in **Table 1**, the hybrid peptide CTP was designed by combining the core functional region of CATH2 with TP5. MS was used to verify the molecular weight of the peptides. The measured molecular weights of the peptides were in agreement with the theoretical values, which suggested that the peptides were successfully synthesized.

**Table 1:** Key physicochemical parameters of parental and hybrid peptides.

Peptides	Sequence	H^a^	Net charge
CATH2	RWGRFLRKIRRFRPKVTITIQGSARF	-0.638	+9
TP5	RKDVT	-1.680	+1
CTP	RWGRFLRKIRRFRRKDVT	-1.483	+8

^a^The mean hydrophobicity (H) is the total hydrophobicity (sum of all residue hydrophobicity indices) divided by the number of residues.

### Cytotoxicity on RAW264.7 Macrophage Cells

**Figure 1:**
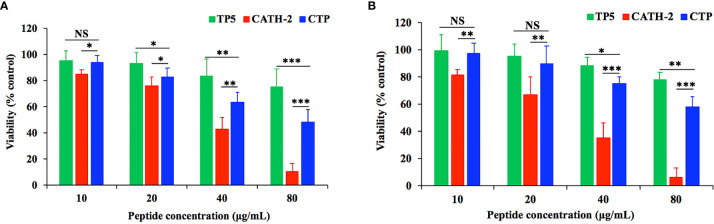
Cell proliferation rates of RAW264.7 cells in the absence or presence of CATH2-TP5 (CTP) and its parental peptides. RAW264.7 cells were pre-seeded in DMEM overnight. RAW264.7 macrophages were treated with the peptides at a series of concentrations ranging from 0 to 80 μg/ml at 37°C and 5% CO_2_ for 24 h **(A)** or 72 h **(B)**. The cells were incubated with CCK-8 solution for 4 h. Finally, the OD value was measured at 450 nm. Data are given as the mean value ± SD from eight biological replicates. NS: p > 0.05, *p ≤ 0.05, **p ≤ 0.01, and ***p ≤ 0.001.

The cytotoxic activity of CTP and its parental peptides towards RAW264.7 macrophages was determined with CCK-8 assays (**Figure 1**). RAW264.7 macrophages were treated with the peptides at a series of concentrations ranging from 0 to 80 μg/ml. CTP exhibited lower cytotoxicity than the parental peptide (CATH2) but higher cytotoxicity than TP5. After incubation with 10 μg/ml peptides for 24 h (**Figure 1A**) and 72 h (**Figure 1B**), the viability of peptide-treated RAW264.7 cells was greater than 80%. These data indicate that at 10 μg/ml all the peptides were minimally cytotoxic to RAW264.7 cells and thus suitable for further anti-inflammatory experiments.

### Anti-Inflammatory Effect of CTP in LPS-Stimulated RAW264.7 Cells

**Figure 2:**
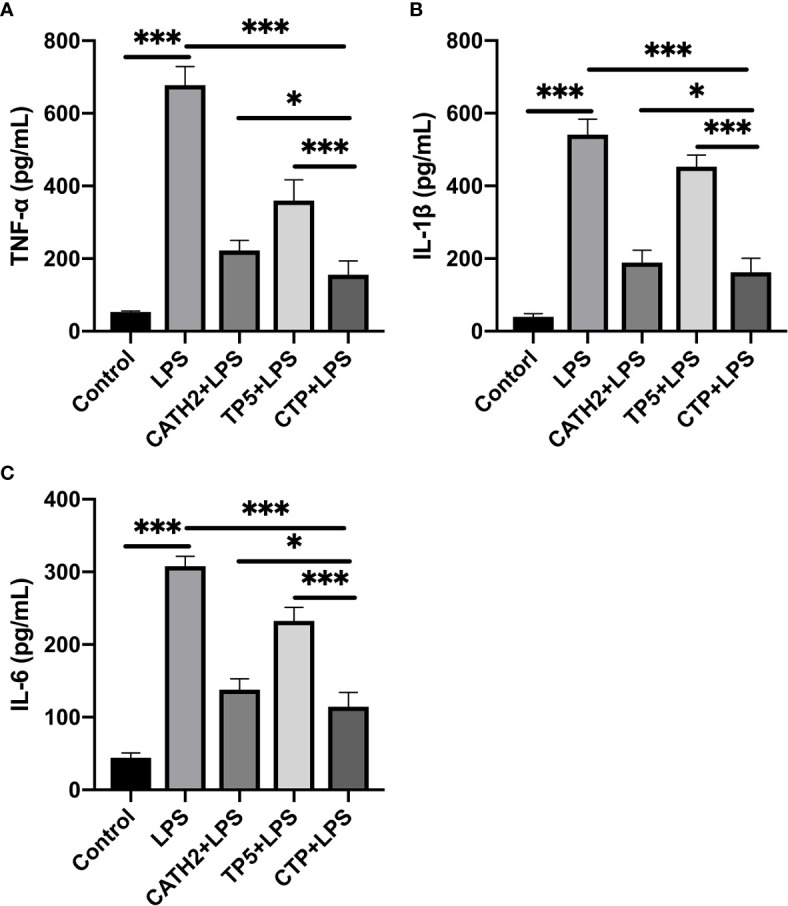
Anti-inflammatory effect of CATH2-TP5 (CTP) in LPS-stimulated RAW264.7 cells. RAW264.7 cells were treated with or without 10 μg/ml peptides for 30 min before the addition of 100 ng/ml LPS and further incubation for 12 h at 37°C. After treatment, the concentrations of TNF-α **(A)**, IL-6 **(B)**, and IL-1β **(C)** in the cell supernatants were assessed using ELISA kits. Data are given as the mean value ± SD from eight biological replicates. *p ≤ 0.05, and ***p ≤ 0.001.

To evaluate the anti-inflammatory effect of CTP and its parental peptides, CATH2 and TP5, RAW264.7 cells were used as a model. The results showed that LPS caused significant elevation of the pro-inflammatory cytokines TNF-α (**Figure 2A**), IL-6 (**Figure 2B**), and IL-1β (**Figure 2C**) compared with untreated cells. As shown in **Figures 2A–C**, all the peptides attenuated the TNF-α, IL-1β, and IL-6 secretion levels. Furthermore, compared with its parental peptides, CTP exerted enhanced inhibitory activity against LPS-induced inflammation.

### CTP Exerts Its Anti-Inflammatory Effect Through LPS Neutralization Activity

**Figure 3:**
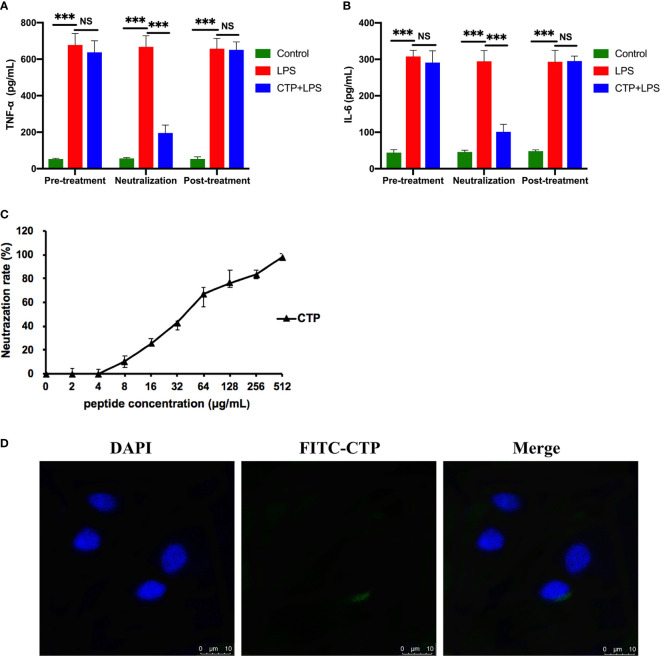
Comprehensive anti-inflammatory activities and cellular localization of CATH2-TP5 (CTP). Time of addition experiments were conducted to determine the anti-inflammatory activity of CTP. RAW264.7 cells were treated with CTP in the time of addition experiments. The cells or lipopolysaccharide (LPS) were treated with CTP at a final concentration of 10 μg/ml under three diverse treatment modes. The concentrations of TNF-α **(A)** and IL-6 **(B)** indicate the anti-inflammatory activity of CTP in each treatment mode. **(C)** *In vitro* LPS neutralization by CTP. LPS neutralization activity of CTP was assessed *in vitro* with a chromogenic Tachypleus Amebocyte Lysate (TAL) assay. **(D)** Cellular localization of CTP in RAW264.7 cells. The peptide was labeled with FITC, and the cellular localization of CTP was assessed by confocal microscopy after incubation for 24 h with RAW264.7 cells. Scale bars: 10 μm. Data are given as the mean value ± SD from three biological replicates. NS: p > 0.05, and ***p ≤ 0.01.

To identify the anti-inflammatory mechanisms of CTP, a time of addition experiment for CTP against LPS-induced inflammation was performed. LPS or RAW 264.7 cells were incubated with CTP at 10 μg/ml for different periods of time, and the anti-inflammatory effects were measured by ELISA. After incubation with LPS, CTP exhibited potent inhibition of pro-inflammatory cytokine release, including TNF-α (**Figure 3A**) and IL-6 (**Figure 3B**), suggesting that CTP might exert its anti-inflammatory activity through interacting with LPS. To verify how CTP works on LPS, an additional test was performed *in vitro.* The results showed that CTP inhibited activation of LPS in a dose-dependent manner (**Figure 3C**). The 50% binding rate value of CTP was approximately 45.32 ± 5.19 μg/ml. In contrast, CTP scarcely reduced the elevation in the pro-inflammatory cytokines TNF-α (**Figure 3A**) and IL-6 (**Figure 3B**) when used to pretreat cells or added to cells after LPS induction. These results suggested that CTP only exerted anti-inflammatory activity when directly interacting with LPS but did not inhibit LPS attachment or affect intracellular anti-inflammatory activity.

Considering the significant difference between the extracellular and intracellular anti-inflammatory activities of CTP, we speculated that CTP may have low cellular uptake in RAW264.7 cells and/or unfavorable intracellular localization. RAW264.7 cells were incubated with FITC-labeled CTP at 10 μg/ml and then examined by confocal microscopy. As shown in **Figure 3D**, FITC-labeled CTP rarely entered RAW264.7 cells, which explained its low intracellular anti-inflammatory activity.

### Design of an Amidation-Modified Peptide Based on the Molecular Template of CTP

CTP was designed and modified to produce a C-terminal amidated derivative peptide, CTP-NH_2_. The structure and molecular weight of CTP-NH_2_ were verified by MS. The HPLC retention time was used to reliably reflect the hydrophobicity of CTP and CTP-NH_2_ in aqueous solution (44). The retention time for CTP and CTP-NH_2_ was 10.94 min and 11.96 min, respectively, indicating that CTP-NH_2_ is more hydrophobic than CTP (**Table 2**).

**Table 2:** Key parameters of CATH2-TP5 (CTP) and its C-terminal amidated derivative peptide CTP-NH_2_.

Peptides	Sequence	Theoretical Mw	Measured Mw	Retention time (min)
CTP	RWGRFLRKIRRFRRKDVT	2445.91	2446.25	10.94
CTP-NH_2_	RWGRFLRKIRRFRRKDVT-NH_2_	2445.91	2445.91	11.96

### Anti-Inflammatory Activities and Cellular Uptake of the CTP Derivative Peptide (CTP-NH_2_)

**Figure 4:**
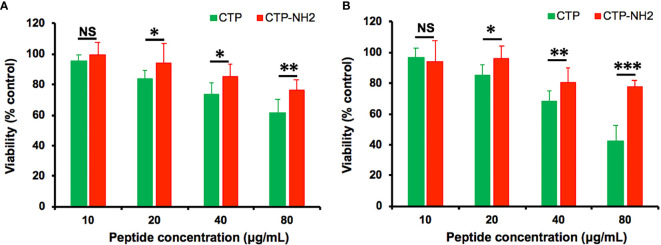
Cytotoxicity, anti-inflammatory activity and intracellular distribution of CTP-NH_2_. The cytotoxicity of CATH2-TP5 (CTP) and CTP-NH_2_ was assessed in RAW264.7 cells using CCK-8 assays. RAW264.7 macrophages were treated with the peptides at a series of concentrations ranging from 0 to 80 μg/ml at 37°C and 5% CO_2_ for 24 h **(A)** or 72 h **(B)**. NS: p > 0.05, *p ≤ 0.05, **p ≤ 0.01, and ***p ≤ 0.001.

The cytotoxicity of the CTP derivative peptide CTP-NH_2_ in RAW264.7 cells was evaluated with CCK-8 assays (**Figures 4A, B**). As the results showed, CTP-NH_2_ exhibited less cytotoxicity than CTP, indicating that CTP-NH_2_ was suitable for subsequent experiments.

**Figure 5:**
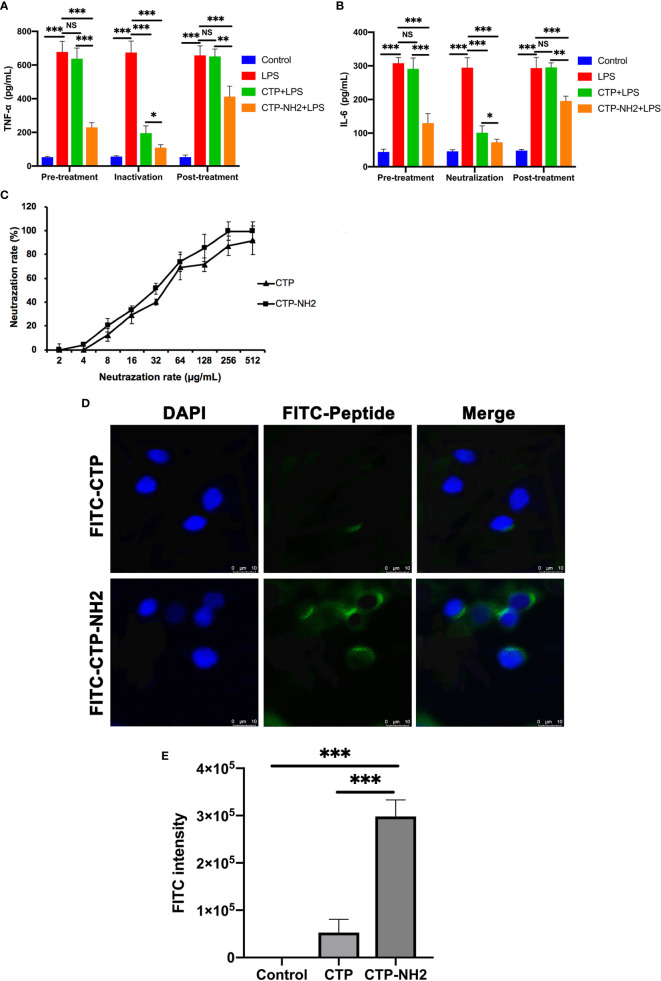
Anti-inflammatory activity and intracellular distribution of CTP-NH_2_. Anti-inflammatory activity assay of CTP-NH_2_ in the time addition experiment. The TNF-α **(A)** and IL-6 **(B)** concentrations show the anti-inflammatory effect of CTP-NH_2_ in each treatment mode. **(C)** *In vitro* LPS neutralization by CTP-NH_2_. The lipopolysaccharide (LPS) neutralization activity of CTP-NH_2_ was assessed *in vitro* using a chromogenic TAL assay. **(D)** Confocal microscopic examination of cellular localization of CTP and CTP-NH_2_. FITC-labeled CTP or CTP-NH_2_ was used to treat RAW264.7 cells for 24 h, and cellular localization was assessed with confocal microscopy. The same image of CTP cellular localization is used in both Figure 5D and Figure 3D. Scale bars: 10 μm. **(E)** Flow cytometry measurement of the cellular uptake of CTP and CTP-NH_2_. The peptides were labeled with FITC and incubated with RAW264.7 macrophages for 24 h, and the average FITC intensity in each cell was determined by flow cytometry. Data are given as the mean value ± SD from at least three biological replicates. NS: p > 0.05, *p ≤ 0.05, **p ≤ 0.01, and ***p ≤ 0.001.

The CTP-NH_2_ modes of action were determined by time of addition experiments as previously described. After incubation with LPS, CTP-NH_2_ exhibited more potency than CTP in inhibiting TNF-α (**Figure 5A**) and IL-6 (**Figure 5B**) secretion. In addition, the LPS neutralization activity of CTP-NH_2_ was stronger than that of CTP (**Figure 5C**). These results indicate that CTP-NH_2_ has greater inhibitory activity against LPS-induced inflammation through neutralization of LPS. Interestingly, CTP-NH_2_ also reduced the concentration of TNF-α (**Figure 5A**) and IL-6 (**Figure 5B**) when used to pretreat cells or added to cells after LPS induction, whereas CTP barely exerted such effects.

To determine whether internalization of the CTP derivative peptide CTP-NH_2_ was promoted by amination of the C-terminus, CTP and CTP-NH_2_ were labeled with FITC and incubated with RAW264.7 cells for 24 h. Afterwards, confocal microscopy and flow cytometry were used to measure the cellular uptake and localization of the peptides. The results showed that CTP-NH_2_ promoted significant cellular uptake and a dispersed distribution compared with CTP (**Figures 5D, E**).

### CTP-NH_2_ Exerted Intracellular Anti-Inflammatory Activity by Binding TLR4/MD-2 and Inhibiting the NF-κB Signaling Pathway

**Figure 6:**
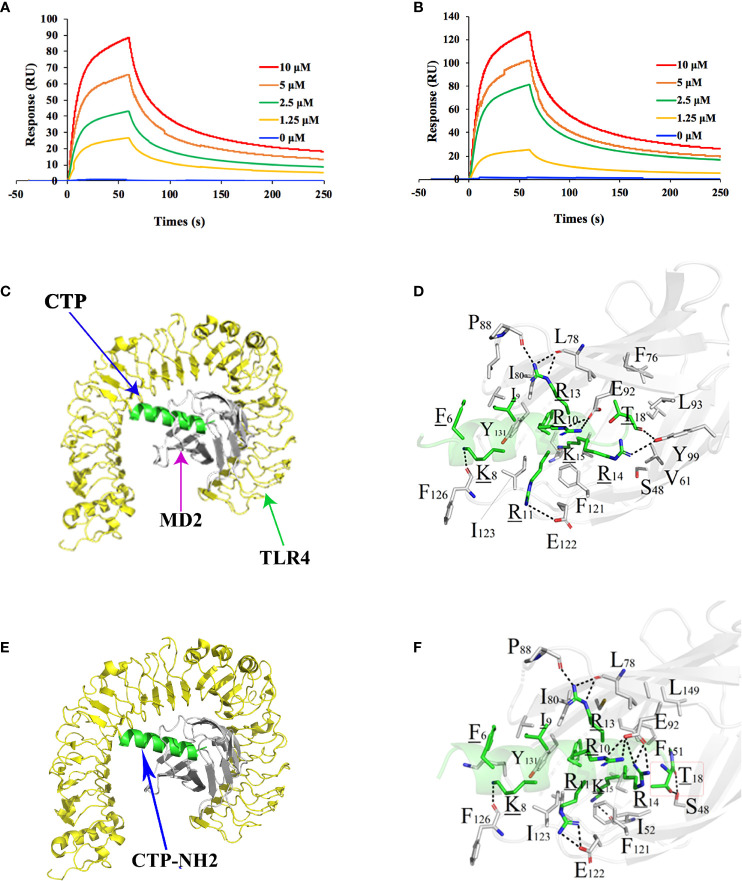
Intracellular anti-inflammatory mechanism of the peptides. The peptide was immobilized on a sensor chip, and the binding of CTP **(A)** and CTP-NH_2_ **(B)** to TLR4/MD-2 complex was analyzed by surface plasmon resonance. Modeled crystal structure of the CTP **(C)** or CTP-NH_2_ **(D)** bound in the hydrophobic “pocket” of MD2 in the TLR4/MD2 complex. Close-up views of the CTP-occupied or CTP-NH_2_-occupied sites in the TLR4/MD2 complex are displayed on the right. The crystal structure of TLR4 is displayed in yellow. The structure of MD-2 is colored in gray, and CTP-NH_2_ is in green. Data are given as the mean value ± SD from at least three biological replicates.

To investigate the intracellular anti-inflammatory mechanism of CTP-NH_2_, binding of CTP-NH_2_ to TLR4/MD-2 was examined *via* an SPR assay. A series of concentrations ranging from 0 to 10 μM were passed over immobilized TLR4/MD-2. The results showed that the peptides binding to the chip-bound protein exhibited a dose-dependent increase (**Figures 6A, B**). The calculated *K*a and *K*d values for CTP-NH_2_ and TLR4/MD-2 binding were 1.65×10^7^ s^–1^ and 1.56×10 M^–1^s^–1^, and the *K*D value was 9.47×10^-1^ μM (**Figure 6B**). Besides, the calculated *K*a and *K*d values for CTP and TLR4/MD-2 binding were 1.34×10^7^ s^–1^ and 1.87×10 M^–1^s^–1^, and the *K*D value was 1.40 μM (**Figure 6A**). These results confirmed that the binding affinity of CTP-NH_2_ for the TLR4/MD-2 receptor was higher than that of CTP for TLR4/MD-2.

Afterwards, to further predict the binding effect of CTP-NH_2_ to the TLR4/MD-2 complex, an MD simulation was performed. A total of 300 snapshots for the peptide-TLR4/MD-2 complex were observed from the last stable 40 ns of the MD simulation. The binding free energy was used to reflect the binding affinity of peptide. As shown in **Table 3**, the binding energy of CTP-NH_2_ was -1,181.25 kJ/mol, which was higher than the binding energy of CTP. In addition, the interface of TLR4/MD-2 that is bound to the peptide (**Figures 6C–F**) was compared to that of LPS (45). The hydrophobic pocket of TLR4/MD-2 for CTP-NH_2_ binding shared the same binding sites and similar residues with those binding LPS (**Figure 6C** and **Table 4**). The interaction between CTP-NH_2_ and TLR4/MD-2 was principally mediated by hydrogen bonds and salt-bridges (**Table 4**). In addition, the TLR4/MD-2- CTP-NH_2_ interaction pair had more hydrogen bonds and salt bridges, and a larger interaction surface area, than those of the TLR4/MD-2-CTP pairs. This is consistent with the SPR results and suggests that CTP-NH_2_ exerts its intracellular anti-inflammatory activity by blocking LPS binding to the TLR4/MD-2 complex.

**Table 3:** Key interaction parameters between the peptide and MD-2.

Interaction Pair	Number of Hydrogen bonds	Number of Salt-bridges	Interaction Surface (Å^2^)	Binding free energy (kj/mol)
MD-2…CTP-NH_2_	14	7	392	-1,181.25
MD-2…CTP	9	3	339	-983.21

**Table 4:** Distance and salt-bridges of binding residues between the peptide and MD-2.

Peptide	Interaction PairMD-2…CTP-NH_2_	Distance (Å)	Number of salt-bridges
CTP	F126…K8	2.89	0
	E122…R11	2.73	1
	Y99…R14	3.12	0
	Y99…T18	3.09	0
	P88…R13	3.25	0
	L78…R13	3.11	0
	E92…R14	2.79	0
	E92…R10	2.67	2
CTP-NH_2_	F126…K8	2.89	0
	E122…R11	2.53	2
	Y99…R14	3.12	0
	Y99…T18	3.09	0
	P88…R13	3.25	0
	L78…R13	3.11	0
	E92…R14	2.49	2
	E92…R10	2.27	3
	S48…T18	2.05	0
	F151…R14	3.31	0
	F151…R10	2.74	0

**Figure 7:**
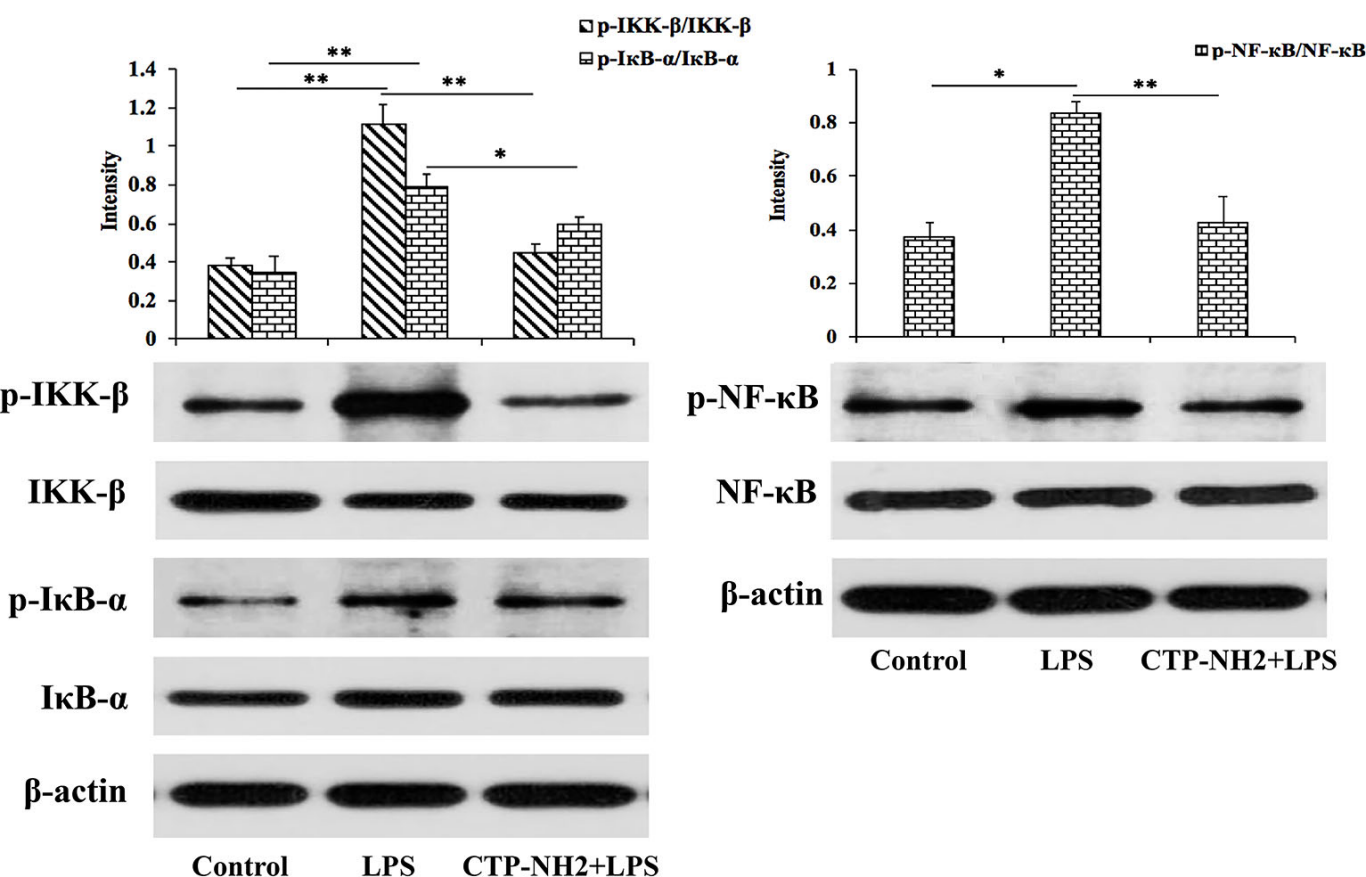
Effect of CTP on the NF-кB signaling pathways. Phosphorylated and total protein levels of IKK-β, IκB-α, NF-кB, and β-actin from serum were measured by western blot analysis. *p ≤ 0.05, and **p ≤ 0.01.

Next, the NF-кB signaling pathway was investigated to determine the intracellular anti-inflammatory mechanism of CTP-NH_2_. LPS significantly increased the phosphorylation of IKK-β, IкB-α, and NF-кB, while cells that were treated with CTP-NH_2_ exhibited dampened levels of IKK-β, IкB-α, and NF-кB (**Figure 7**). These results suggest that the intracellular anti-inflammatory effect of CTP-NH_2_ on the NF-κB signaling pathway plays a crucial role in the process by which CTP-NH_2_ modulates LPS-induced inflammation.

### The Protective Effects of CTP-NH2 Against LPS-Induced Sepsis

**Figure 8:**
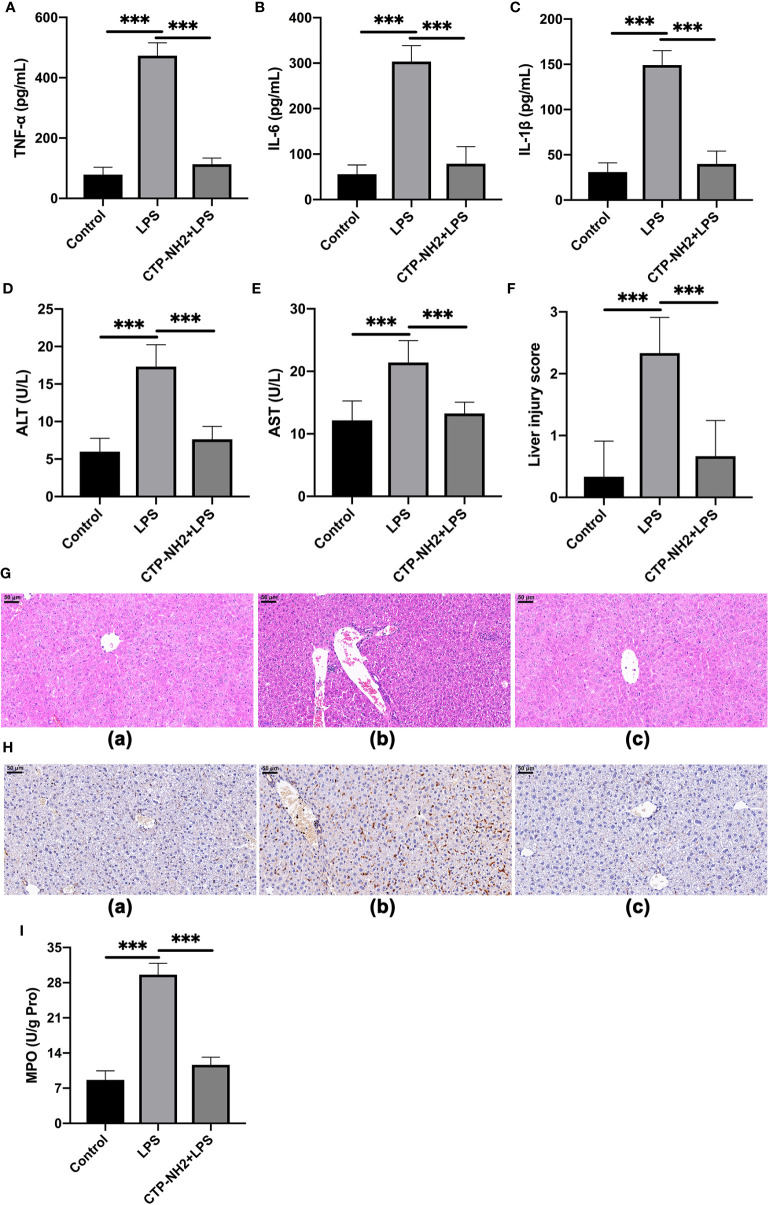
The protective effects of CTP-NH2 against LPS-induced sepsis in mice. (10 mg/kg) were injected into the mice once daily for 6 days, whereas the control and LPS-treated groups were injected with an equal volume of sterile saline. On day 6, mice in the LPS and peptide-pretreatment groups were injected with LPS (10 mg/kg) 1 h after the peptide or saline treatment. The control group was injected with an equal volume of saline. ELISAs were performed to detect TNF-α **(A)**, IL-6 **(B)**, and IL-1β **(C)** in serum. The expression of alanine amino transferase (ALT) **(D)** and aspartate transaminase (AST) **(E)** in serum. **(F)** The effect of CTP-NH2 on liver injury scores. **(G)** Representative H&E-stained sections from the (a) control, (b) LPS, and (c) CTP-NH2 + LPS groups. Bar, 50 μm. **(H)** Representative images of CD177+ cells. Bar, 50 μm. Formalin-fixed, paraffin-embedded, 5-mm cross-sections were stained with a primary Ab against CD177+. (a) control, (b) LPS, and (c) CTP-NH2 + LPS groups. The enzymatic activity of MPO was measured **(I)**. Data are given as the mean value ± SD from at least three biological replicates. ***p ≤ 0.001.

To characterize the inhibitory effect of CTP-NH_2_ against LPS-induced sepsis, the concentrations of the inflammatory markers TNF-α, IL-6, and IL-1β in mouse serum were quantified *via* ELISA. Compared with the control group, LPS challenge led to significant increased levels of TNF-α (**Figure 8A**), IL-6 (**Figure 8B**), and IL-1β (**Figure 8C**) in the serum of mice, whereas the CTP-NH_2_-pretreated group showed significantly decreased levels of TNF-α, IL-6, andIL-1β compared with those in the LPS-treated group.

LPS clearly caused an increase in ALT and AST, markers of liver function, whereas CTP-NH_2_ significantly attenuated these effects (**Figures 8D, E**). Furthermore, compared with the control group, LPS caused considerable tissue injury, with disturbed hepatic architecture, extensive hemorrhage, hepatocyte necrosis and inflammatory cell infiltration (**Figure 8G**). By contrast, the severity of liver injury was attenuated by CTP-NH_2_ pretreatment (**Figure 8G**). These protective effects were confirmed by liver injury score analysis (**Figure 8F**).

Moreover, immunohistochemistry results showed that LPS triggered increased infiltration of CD177^+^ neutrophils into the liver lesion area compared with the control (**Figure 8H**). However, the infiltration of neutrophils was significantly lower in the CTP-NH_2_-pretreated group than in the LPS-treated group. As an index of neutrophil infiltration and inflammation (46), the activity of MPO in the mouse liver was evaluated by ELISA. Consistent with the immunohistochemistry results, the MPO activity was markedly increased in LPS-treated mice, but pretreatment with CTP-NH_2_ significantly reduced this effect (**Figure 8I**).

## Discussion

In the recent years, many anti-inflammatory peptides have been discovered or designed, and some have exerted potential LPS neutralization activity (7, 47, 48). However, their development has been weakened by several concerns, including potential cytotoxicity (22) and weak physiological stability and poor anti-inflammatory activity (49). To obtain a novel anti-inflammatory peptide with increased activity but minimal cytotoxicity, hybridization has been proposed (50, 51). Our group has completed several studies of hybrid anti-inflammatory peptide designs that can improve the anti-inflammatory activity and reduce the undesirable cytotoxic effects of native peptides (52, 53). Anti-inflammatory experiments showed that the new designed peptides can inhibit LPS-induced inflammation by neutralizing LPS, binding to the TLR4/MD-2 complex or inhibiting the NF-кB signaling pathway (52, 53).

In this study, we designed a hybrid peptide by combining the active center of CATH2 (1–13) (14) with TP5. The anti-inflammatory activities of the hybrid peptide and its parental peptides were verified by ELISAs in RAW264.7 cells. CTP, the new designed peptide, markedly reduced the levels of TNF-α, IL-6, and IL-1β compared with its parental peptides, CATH2 and TP5. The cytotoxicity of CTP was further tested, and the results showed that CTP had lower cytotoxicity than its parental peptide (CATH2). In addition, CTP is minimally toxic at a concentration of 10 μg/ml. Unfortunately, CTP scarcely exerted inhibition of TNF-α and IL-6 secretion when preincubated with cells before LPS induction or added to cells after LPS induction. Furthermore, CTP only exhibited inhibition when added to the cells being incubated with LPS. Confocal microscopy and flow cytometry analyses showed that CTP exhibited extremely poor cellular uptake because no visible FITC-labeled CTP was detected in the incubated cells.

To overcome the difficulty of peptide access and entry into the cell, various methods have been employed. For instance, introduction of histidine residues (27, 28) or addition of D-amino acids (29, 30) may enhance peptide transmembrane delivery. Furthermore, peptide hydrophobicity is required for enhanced cellular uptake (31, 32) and C-terminal amidation has been reported to enhance the hydrophobicity of peptides (33). These studies suggested that C-terminal amidation can be used to enhance cellular uptake and anti-inflammatory activity (54, 55).

In our study, CTP was designed and modified to produce a C-terminal amidated derivative peptide. The HPLC retention time showed that the derivative peptide (CTP-NH_2_) exhibited stronger hydrophobicity than CTP. In addition, the derivative peptide CTP-NH_2_ showed improved anti-inflammatory activities and decreased cytotoxicity. TNF-α and IL-6 secretion analysis showed that CTP-NH_2_ exhibited greater inhibitory activity against LPS-induced inflammation when incubated with LPS in cell culture medium compared with CTP, which may be due to the stronger LPS neutralization activity of CTP-NH_2_. Furthermore, it is worth noting that CTP-NH_2_ also reduced the concentration of TNF-α and IL-6 when used to pretreat cells or added to cells after LPS induction, whereas CTP barely exerted such effects. Flow cytometry and confocal microscopy results showed that CTP-NH_2_ exhibited considerable cellular uptake and a dispersed distribution, which may explain its anti-inflammatory activity. Therefore, these results indicate that the C-terminal amidation of CTP molecules can enhance hydrophobicity and thus overcome barriers in cellular uptake and improve anti-inflammatory activity.

To identify the mechanisms of the observed anti-inflammatory effects when cells were pretreated with the peptide or the peptide was added to cells after LPS induction, a comprehensive and detailed analysis was performed. Toll-like receptor (TLR) is endowed with the capacity to sense conserved molecular patterns on microbial pathogens and mount immune responses in host defense (56, 57). TLR4 is primarily activated by LPS recognition through an accessory protein-MD-2 (58). Hence, blocking TLR4/MD-2 is a potential mechanism for attenuation of the LPS-induced inflammatory response (59–61). To investigate the ability of CTP-NH_2_ to bind to the TLR4/MD-2 complex, SPR binding assays were performed. The SPR results confirmed that CTP-NH_2_ could effectively bind to TLR4/MD-2. Consistently, MD simulation showed that CTP-NH_2_ could bind to the hydrophobic pocket of MD-2, which partially overlaps with the LPS binding site on MD-2 (45). Thus, the results suggest that CTP-NH_2_ confers its anti-inflammatory activity through blocking LPS binding to the TLR4/MD-2 complex. Furthermore, LPS is a strong activator of the NF-κB signaling pathway though its interaction with TLR4 (62). Thus, NF-κB plays a crucial role in host defenses through regulation of inflammatory gene expression (63). In the present study, the expression of the major proteins involved in the NF-κB pathway were detected by western blotting to elucidate the anti-inflammatory mechanism of CTP-NH_2._ The results showed that CTP-NH_2_ effectively inhibited activation of the NF-κB pathway by decreasing the phosphorylation of IKK-β, IкB-α, and NF-кB.

The *in vivo* anti-inflammatory activities of CTP-NH_2_ were also evaluated in an LPS-induced murine model of sepsis. LPS, a major endotoxin, has been considered a major cause of sepsis (3). In addition, 20 mg/kg LPS has been reported to induce sepsis *in vivo*, which can cause excessive inflammation and organ failure, such as in liver tissue (64). Consistent with previous studies, the present study showed that the levels of TNF-α, IL-6, and IL-1β were markedly increased in LPS-treated mice, while pretreatment with CTP-NH_2_ efficiently reduced this effect. Liver injury is considered one of the most serious health problems in the world, can result from diverse etiologies and is associated with high mortality (65). In this study, LPS induced liver injury, with obvious changes in biochemical and histopathological parameters. As biochemical markers of liver injury, AST and ALT are used to reflect liver injury during clinical trials. The present study showed that AST and ALT were markedly increased by LPS. However, CTP-NH_2_ effectively decreased the AST and ALT levels. In addition, histological analysis showed that CTP-NH_2_ repaired hemorrhage and cellular necrosis in the liver. The infiltration of activated neutrophils, one of the most representative histological features observed in liver inflammation (66), was significantly increased in LPS-treated mice. However, pretreatment with CTP-NH_2_ prevented infiltration of activated neutrophils in the liver. Consistent with this, liver MPO activity, an index of neutrophil infiltration and inflammation, was significantly increased in LPS-treated mice, but pretreatment with CTP-NH_2_ significantly reduced this effect. Collectively, these results indicate that CTP-NH_2_ can efficiently prevent LPS-induced sepsis in mice.

## Conclusion

**Figure 9:**
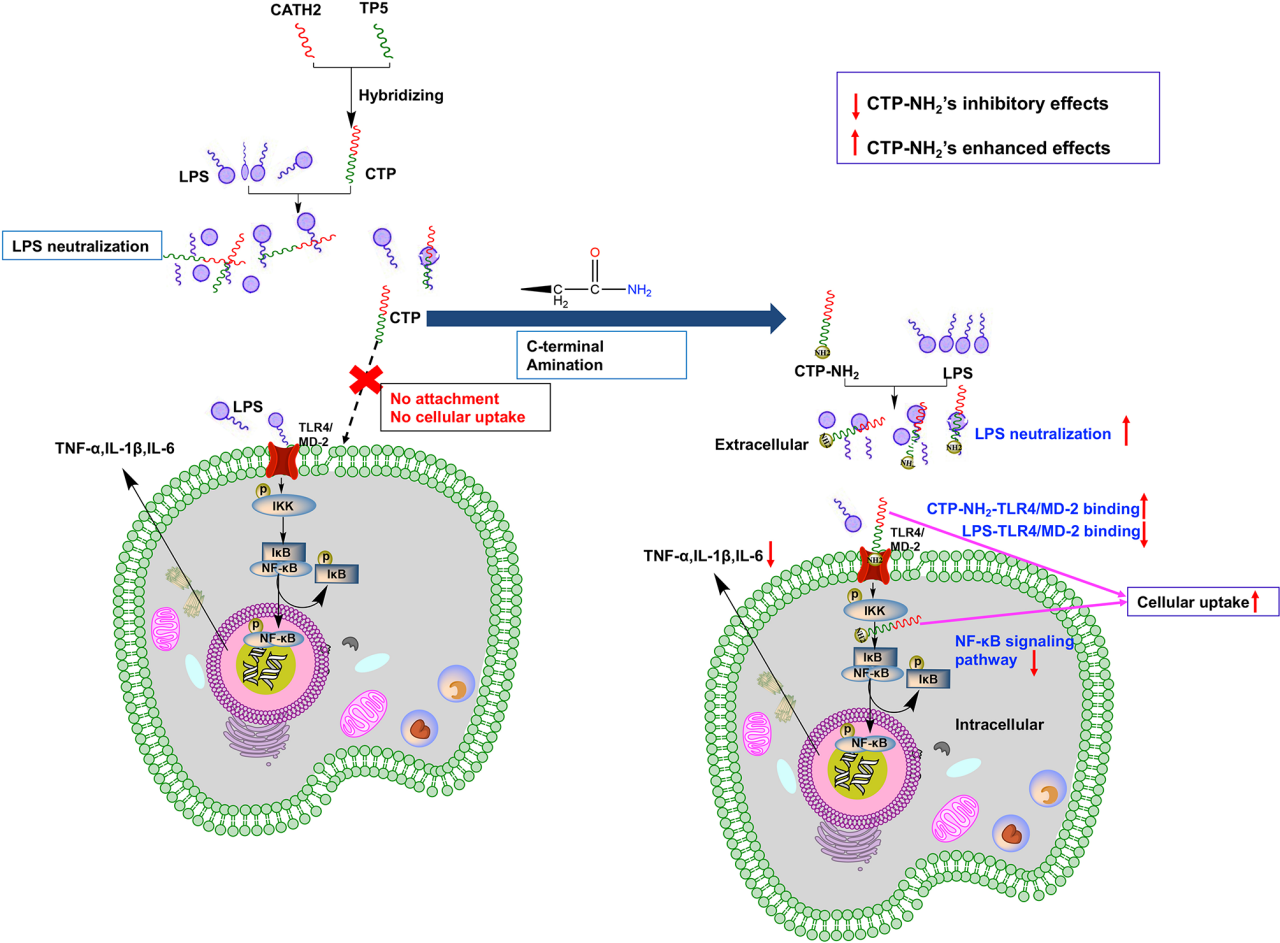
Schematic illustration of design and development of the novel hybrid peptide, CTP-NH_2_, for the treatment of LPS-induced inflammation.

The successful design and modification of CTP may provide an avenue to modify previously discovered peptides to improve their anti-inflammatory properties or design novel active peptide agents with excellent cellular uptake and anti-inflammatory activities (**Figure 9**). In addition, our study revealed that the anti-inflammatory effects of CTP-NH_2_ associated with LPS neutralization, binding activity on the TLR4/MD-2 complex, and inhibition of the NF-кB signaling pathway.

## Data Availability Statement

The original contributions presented in the study are included in the article/supplementary material. Further inquiries can be directed to the corresponding author.

## Ethics Statement 

The animal study was reviewed and approved by Institutional Animal Care and Use Committee of China Agricultural University.

## Author Contributions

LZ, XW, RZ, MK, and DS conceived the project and designed the experiments. LZ, XW, BA, and HG conducted experiments. LZ and MK wrote the manuscript and analyzed data. All authors read and commented on the manuscript. All authors contributed to the article and approved the submitted version.

## Funding

This work was supported by the National Key Research and Development Program of China (Project No. 2018YFD0500600), the National Natural Science Foundation of China (NSFC, 31572442), and the National Natural Science Foundation of China (NSFC, 31272476).

## Conflict of Interest

The authors declare that the research was conducted in the absence of any commercial or financial relationships that could be construed as a potential conflict of interest.

## Abbreviations

CATH2, cathelicidin 2; CATH2-TP5, CTP; CCK-8, Cell Counting Kit-8; CE TAL, Chromogenic End-point Tachypleus Amebocyte Lysate; DAB, 3, 3’diaminobenzidine; DMEM, Dulbecco’s modified Eagle’s medium; FBS, fetal bovine serum; HRP, horseradish peroxidase; H&E, hematoxylin-eosin; IL-6, interleukin-6; LPS, lipopolysaccharide; MD, molecular dynamics; MM-PBSA, Poisson-Boltzmann accessible surface area; MPO, myeloperoxidase; NF-кB, nuclear factor-kappa B; NPT, number, pressure, and temperature; NVT, number, volume, and temperature; SPF, specific pathogen free; TLR4/MD-2, Toll-like receptor 4/myeloid differentiation factor 2; TNF-α, tumor necrosis factor-alpha; TP5, thymopentin.
